# High-Resolution Ultrasound Platform for Infant Meningitis Detection: An In Vitro Demonstration

**DOI:** 10.3390/s24154768

**Published:** 2024-07-23

**Authors:** Manuel Navarrete, David Castells-Rufas, Hassane Baghdad Kichou, Guillermo Navarro-Patron, Javier Jimenez, Jordi Carrabina

**Affiliations:** 1 Kriba, Barcelona Science Park, 08028 Barcelona, Spain; hassane.kichou@kriba.ai (H.B.K.); guillermo.navarro@kriba.ai (G.N.-P.); javier.jimenez@newborn.solutions (J.J.); 2 Department of Microelectronics and Electronic Systems, Autonomous University of Barcelona, 08193 Barcelona, Spain; david.castells@uab.cat (D.C.-R.); jordi.carrabina@uab.cat (J.C.)

**Keywords:** ultrasound, white blood cells, high resolution, detection, microscopy, cerebrospinal fluid, meningitis

## Abstract

Infant meningitis remains a severe burden on global health, particularly for young infants. Traditional ultrasound imaging techniques are limited in spatial resolution to visualize white blood cells (WBCs) in the cerebrospinal fluid (CSF), which is considered a well-established marker for meningitis detection. This work presents a novel platform that uses high-resolution ultrasound to detect the backscatter signals from microscopic CSF WBCs through the anterior fontanelle of neonates and young infants. The whole system was built around a custom probe that allows for a 20 MHz focused transducer to be mechanically controlled to map the area of interest in the CSF. Data processing can be performed internally in the device without the need to extract the images for further analysis. The in vitro feasibility of the proposed solution was evaluated in imaging 7 μm particle suspensions at different concentrations relevant to meningitis diagnosis ranging from 7- to 646-particles (pp)/μL. The experimental tests were conducted from a simple setup using a sample container to a more realistic setup based on an anatomical phantom of the neonatal head. The results show high-quality images, where 7 μm particles can be resolved for the different concentrations.

## 1. Introduction

Meningitis is a severe disease with a high mortality rate, often causing serious long-term complications. Despite advances in diagnosis, treatment, and vaccination [1], in 2016, there were 2.82 million cases of meningitis worldwide, with 318,000 related deaths [2]. The highest incidence of meningitis in the world is in sub-Saharan Africa, known as the African Meningitis Belt stretching from Ethiopia to Senegal [3,4]. Traditional methods to diagnose meningitis typically involve a clinical examination followed by a lumbar puncture. This procedure is considered the gold standard for diagnosing meningitis [5]. This implies inserting a thin needle into the lower back to collect a sample of cerebrospinal fluid. Analysis of the CSF can reveal the presence of infection by observing an increase in white blood cells [6] and other markers that help identify the cause and severity of meningitis. Other diagnostic methods, such as blood tests and imaging studies, may be used in conjunction with a lumbar puncture to support the diagnosis and guide treatment [3].

Meningitis suspicion is challenging due to the unspecific symptoms associated with the infection, such as fever, headache, and neck stiffness, among others, which can also be caused by other conditions [7]. Particularly, signs and symptoms are less evident in neonates and infants [3]. This is why clinical history and physical evaluation alone are generally insufficient to confirm or exclude the diagnosis. Lumbar puncture to draw a sample of cerebrospinal fluid is an essential and systematic investigation by protocol in the suspected patient but difficult to perform and potentially hazardous for a sick child [8]. Although meningitis affects all ages, young children are most at risk. Particularly, infants under the age of 1 year are at a particularly high risk for meningitis [9,10], especially newborns in the first month of life because their immune system are not fully developed [11]. Therefore, since they are most susceptible to infections, special attention should be paid to this age group.

All these challenges demand practical solutions that enable alternative ways for direct mapping of the CSF in infants. The assessment of white blood cell concentration in the CSF using minimally invasive or non-invasive approaches would provide valuable information while complementing the limitations of lumbar puncture or other invasive methods. Innocuous and non-invasive ultrasound has been used for the characterization of biological tissues and is presented as a suitable tool for the measurement of cell concentrations in serous body fluids, specifically in the CSF [12,13,14,15]. As usually performed in cranial ultrasound (CUS), images can be obtained through the anterior fontanelle of the neonate to take advantage of this acoustic window [16,17]. This way, the skull bone is avoided, which is the main obstacle for high-resolution ultrasound brain imaging [18]. Such method has the potential to achieve real-time measurements while applying minimal stress to the patient under evaluation.

### 1.1. Related Work

Improving the resolution of ultrasound imaging can be obtained by increasing the frequency, but there is an inherent compromise between penetration and resolution which limits spatial resolution by diffraction in a far-field regime [19]. Beyond increasing the frequency to improve the resolution, recent developments in optical microscopy have inspired analogous approaches to overcome the half-wavelength limit in ultrasonic imaging [20]. Because of its relevance, we will review the different ultrasound-based approaches for super-resolution.

Ultrasound super-resolution imaging comprises several techniques, using high-frequency acoustic waves, that can distinguish objects or structures closer than the classical diffraction limit. These techniques include ultrasound localization microscopy, super-resolution fluctuation imaging, structured illumination, or near-field approaches [21].

Ultrasound localization microscopy is based on ultrafast imaging for tracking the flow of contrast agents enabling one to overcome the diffraction-limited resolution of vascular structures down to microscopic resolutions [20,22]. This technique requires the injection of contrast agents into the body: in this case, gas microbubbles of a few micrometers in size. Because ultrasound is so sensitive to these pockets of gas, they can be spatially separated to yield precise positioning. Through a large number of acquired images, the sub-wavelength location of these many individual sources is accumulated to recreate a super-resolved composite image. These techniques are currently limited by low temporal resolution and long acquisition times [21]. Ultrafast imaging by using plane-wave emissions rather than line-by-line pulse echo has increased frame rates to thousands of frames per second, enhancing blood flow sensitivity by more than an order of magnitude. This increase in sensitivity led eventually to ultrasound-based brain functional imaging using the detection of small blood volume changes as a surrogate for brain activity [23].

Also inspired in optics, super-resolution fluctuation imaging exploits the high-order statistics of temporal fluctuations in the ultrasound signal to reduce the size of the point-spread function of the images. Adaptations of this technique have also been proposed using ultrafast tracking of contrast agents (microbubbles) to improve resolution [24,25]. This method does not require the localization of individual emitters; therefore, a smaller number of images are required. However, this process is limited to an improvement of resolution to a factor of 2 with respect to the resolution limit of the system. These techniques are now being applied pre-clinically and clinically for the imaging of the microvasculature of the brain, kidney, skin, tumors, and lymph nodes [21]. However, these methods require an invasive procedure to inject the contrast agents into the body.

Structured illumination microscopy is another optical method that was extended to increase the spatial resolution of ultrasound imaging [26]. The emission pattern is manipulated to encode the high spatial frequencies into the observed image through aliasing. Using post-processing techniques for the reconstruction of a super-resolved image enhances resolution by a factor of 2 with respect to the resolution limit of the system. This method can be employed to improve vascular visualization.

The diffraction limit is applicable in the far-field regime. In the near field, the spatial resolution is proportional to the distance with respect to the object and independent of wavelength. Less than a few wavelengths away from a probe, sub-wavelength sources can be differentiated at the 10 to 100 nanometer scale [27]. However, if the target were organs in the body, often at hundreds of wavelengths from the probe, near-field super-resolution techniques are difficult to apply. In recent years, the introduction of metamaterials has enabled super-focusing in the near field, achieving resolutions close to one-third of the wavelength, which is beyond the diffraction limit [28]. Another way to overcome the inherent limits of spatial resolution is to combine ultrasound and other waves of a different nature [29]. For example, a combined imaging method of ultrasound and photoacoustic has been demonstrated to measure single leukocytes with micrometer resolution [30].

From the perspective of the analysis of the acoustic properties of cells, studies have been previously proposed to characterize cell structure changes in cell ensembles using high-frequency ultrasound (20, 40 MHz) [31]. From exploiting alterations in acoustic properties, such as spectral slope, a variation in cell morphologies was detected. Due to problems with localization and low signal strength of cells, it is difficult to measure acoustic scattering from single cells, and thus to achieve a comprehensive understanding of the scattering interaction at the cellular level. For this purpose, backscatter measurements from microspheres and different types of individual cells have been performed and compared with proposed models of acoustic scattering from single cells [32,33]. Studies on cell suspensions (with typical sizes in the range 6–30 μm at different concentrations) using ultrasound systems above 20 MHz have been conducted from the echoes backscattered by single cells [34,35]. More recently, it has been shown that high-frequency ultrasound (20–75 MHz) can be used to measure the ultrasound echoes backscattered by cells to detect low CSF WBC concentrations in vitro [13,14].

Despite all these advances, to the best of our knowledge, no practical solution based on ultrasound has been developed which can be employed for directly imaging the white blood cells through the fontanelle of infants. A microscopic analysis of the CSF remains limited due, in part, to the lack of non-invasive imaging solutions providing microscopic resolutions.

### 1.2. Promising New Solution for Early Detection of Infant Meningitis

**Figure 1:**
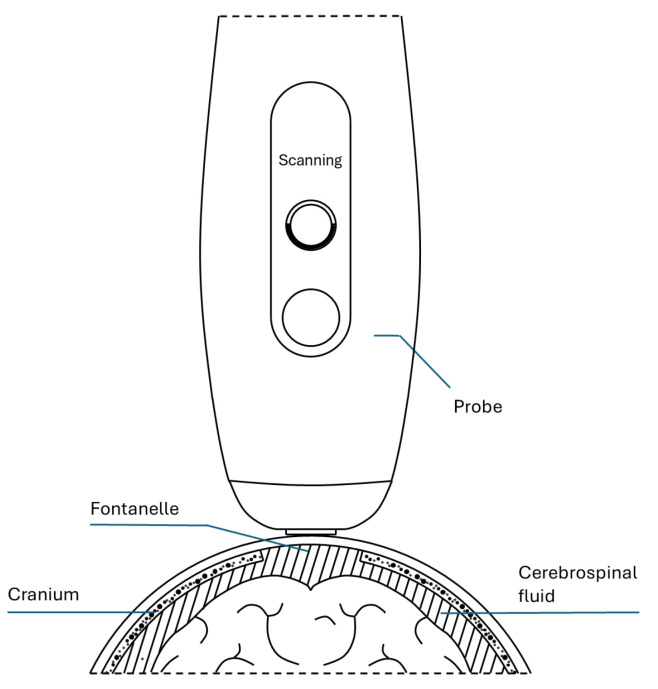
Cross-sectional cut of the proposed hand-held ultrasonic device to detect meningitis.

The assessment of the white blood cell concentration in the CSF is key to the diagnosis of meningitis. The proposed solution consist of a high-frequency ultrasound-based instrumentation capable of detecting backscatter signals from WBC in the CSF through the anterior fontanelle of neonates and young infants (see Figure 1). Considering the different anatomical characteristics of each patient, a mechanism is provided to locate a suitable view of the CSF. From this wide overview, it is possible to zoom in (increasing lateral resolution) on a region of interest where the respective WBC concentration can be evaluated. This solution builds on and extends a previously proposed method about an application of ultrasound imaging for in vitro assessment of WBC concentration from the measurement of the integrated backscatter coefficient of cells [13]. This original proposal was based on a commercial platform that was adapted to the particular area of study.

In this context, this article focuses on the development of a complete portable ultrasound platform that can offer new possibilities for ultrasound research in this field by increasing the resolution to allow for the detection of WBC in the CSF. This requires the efficient integration of hardware and software components into a resource-constrained platform to enable accurate CSF imaging. Furthermore, initial attempts have shown that this programmable platform can be adapted to fit specific requirements, allowing for rapid introduction and in vivo testing of the application, which is beyond the scope of this work.

## 2. Challenges in CSF White Blood Cells Detection through the Fontanelle of Infants Using Conventional Ultrasound Imaging

White blood cells are a challenging target to identify within the CSF using ultrasound due to their small size and location. The dimensions of the WBCs are reported to fall typically between 9 and 18 μm [36]. Due to their small size, even at high ultrasound frequencies (20 to 75 MHz), cells are smaller than the wavelength of the incident sound wave (75 to 20 μm) [32]. With sizes that can even go down to 5 μm, the intensity waves backscattered by these small cells are increasingly low. As an example, at a frequency of 50 MHz, the energy of the acoustic wave backscattered by a 7 μm diameter cell would be approximately 15 times less than for the same type of cell but with twice the diameter [12]. Cells typically have acoustic properties similar to the surrounding tissue, leading to minimal acoustic impedance mismatch [12]. This low contrast in acoustic properties makes it difficult to differentiate cells from the background. Additionally, ultrasound waves are attenuated as they travel through tissue, which can further reduce the signal strength. In general, not only because of their small size, but also due to their weakly scattering nature, the scattered sound from individual cells may be too low to be detected above the background noise of the system [32], resulting in a relatively low signal-to-noise ratio (SNR).

### 2.1. Depth Attenuation

When ultrasound waves cross through the body before reaching the region of interest in the CSF, the intensity of the beam is attenuated. This attenuation depends on both the energy absorbed by the medium and the energy dispersed by interfaces or tissue inhomogeneities [19]. The fontanelle tissue constitutes the major hurdle affecting the quality of the ultrasound images on this application. Tissue may have different characteristics depending on the patient, which increases the variability of backscattered echo energy by cells among different patients [14]. For cells that are small compared to the working wavelength, energy is scattered in many directions until being eventually absorbed due to particle vibration and heat production [37].

Attenuation at a given distance varies with ultrasound frequency. A high-frequency wave is attenuated more than a low-frequency wave. However, since higher frequencies allow for more detail to be resolved, there is an inherent trade-off between resolution and penetration for different imaging applications [38]. Resolution becomes essential when imaging small targets such as CSF white blood cells.

### 2.2. Resolution

Both spatial and temporal resolution must be taken into account. Spatial resolution is further divided into axial and lateral resolutions. Axial resolution is the minimum distance where two reflectors located along the direction of the ultrasound beam can be distinguished. As shown in Equation (1), axial resolution ($\Delta_{axial}$) is dependent on the sound wave pulse length, referred to as spatial pulse length (SPL). Specifically, axial resolution is equal to half the spatial pulse length. Spatial pulse length is the product of the number of cycles (ε) in an ultrasound pulse and the wavelength (λ) [39].

(1) $$\Delta_{axial}=\frac{SPL}{2}=\frac{\varepsilon \lambda }{2}$$

The number of cycles is caused by damping of the crystals after excitation. High damping reduces the number of cycles of the pulse and, thus, reduces the SPL. The pulse wavelength is given by the operating frequency of the transducer. High-frequency transducers generate pulses of short wavelength [40]. Due to diffraction, the resolution of ultrasound imaging is theoretically limited to about half a wavelength. Below this limit, the echoes from many scatterers overlap and become no longer distinguishable. This leads to the fundamental trade-off between resolution and penetration depth, as attenuation increases with frequency [38]. In general, conventional ultrasound imaging techniques cannot overcome the axial-resolution limit determined by the wavelength of the transmitted pulse.

Lateral resolution is the minimum distance at which two reflectors located perpendicularly to the direction of the ultrasound beam can be distinguished. Lateral resolution depends on the width of the beam and the frequency of the sound waves [41]. A narrower beam width provides higher lateral resolution. The beam width can be reduced by focusing the sound waves as they are produced by the transducer. Beam focusing can be performed by using a piezoelectric focused transducer to produce an ultrasound pressure field, typically concentrated within an ellipsoid region [42]. Lateral resolution is higher in the focal zone, where the beam width is narrower and can be assumed to be planar. Lateral resolution is also affected by the frequency of the sound wave, as higher frequencies tend to improve resolution [37]. Thus, the resonant frequency of the crystal, the design of the probe, and the rest of the system are limited by this trade-off between resolution and penetration, restricting the requirements to a specific application.

Temporal resolution is the time elapsed from one frame to the next. It represents the ability to distinguish two events in time. Hence, it is essential for imaging rapidly moving structures. Time resolution becomes less crucial for the more static structures [43].

## 3. System Design and Implementation

In this section, we delve into the design and development of the proposed solution. We start by the key design requirements that must be considered. The hardware architecture of the system is then detailed, highlighting its components and their functions. Next, we explain how the system works to achieve the desired functionality. Finally, we describe some implementation details, focusing on the software solution in relation to specific constraints.

### 3.1. Design Considerations

The choice of transducer frequency is an essential consideration to ideal ultrasound image acquisition. Since axial and lateral resolution is improved as frequency increases, higher frequency transducers are usually selected for resolving small objects in the ultrasound image. As mentioned in the previous section, one limitation of using high frequencies is that as the frequency increases, the penetration of the sound wave decreases. By recognizing this intrinsic compromise, the user can choose the highest frequency transducer that penetrates to the depth of the target structure. For example, a 5–15 MHz transducer should be selected to analyze depths in the range of 15 to 4 cm [41]. A 15–20 MHz transducer may image depths of only 4 to 3 cm. Higher frequencies like 30–40 MHz may penetrate only 2 to 1 cm [43].

Specifically, an increasing backscattering amplitude was found as the frequency rose up to 30 MHz for human acute myeloid leukemia cells (11.5 μm diameter) [33]. Also, considering the downward shift in the center frequency of ultrasound pulses that propagate through biological tissues, as a result of the attenuation dependence on the frequency, the optimum operating frequency could be below 30 MHz for similarly sized cells [14].

On the other side, the pooled mean size of anterior fontanelle is around 2.58 cm [44]. Thus, there is a physical limitation to the size of this acoustic window that allows for access to the CSF while avoiding the skull bone. Finally, a mechanically driven single-element transducer that operates at 20 MHz was employed considering these restrictions. This frequency compensates for the loss of cellular backscatter energy with lower attenuations of the ultrasound beam as it travels through tissue [14]. This is a custom focused transducer with an f-number between 1.4 and 1.8. A focused transducer was selected to enhance the amount of energy reflected from the cells. As mentioned above, the temporal resolution is not as critical when imaging target structures that are relatively static, such as the CSF. However, a high acquisition rate of scan lines is essential to ensure lateral coherence within the image.

### 3.2. Architecture and Components

**Figure 2:**
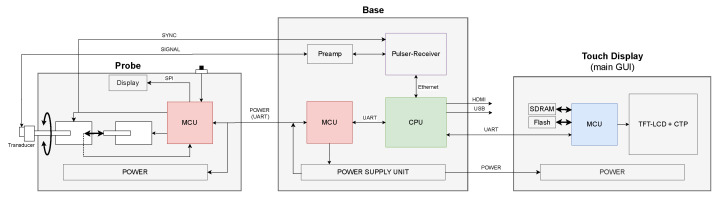
Block diagram of the system.

The proposed architecture is based on a heterogeneous computing platform, consisting of different modules connected to execute the different tasks of the system. Each subsystem is based on a commercially available processing unit selected to maximize its performance. Figure 2 shows a block diagram of the system with colored processing units.

CPU: The NVIDIA Jetson Nano acts as the main computational element of the platform, controlling and coordinating the different tasks implemented in each subsystem. The CPU controls the functions of the ultrasound pulser-receiver, including tasks such as data acquisition, parameter configuration, digital signal processing, image composition as well as user interface, storage, plotting, and data visualization. The Jetson Nano is the computational core of the system, facilitating data exchange and communication between the different components. This application provides a secondary user interface for system configuration, monitoring, and extended interaction. Tools for analyzing and obtaining quantitative measurements from the ultrasound signal are also available.

Probe: This module, based on a microcontroller unit (MCU), manages all the probe peripherals according to the instructions received from the Jetson Nano. A button on the probe starts the scanning process. The mechanical motion of the structure that holds the transducer is controlled by two stepper motors allowing for positioning (in depth) and moving the transducer during acquisition. A photo-detector sets the safe limits and identifies reference positions within the direction of penetration of the transducer. Complementary user information is shown on a local small display.

Base MCU: Another MCU acts as a communication bridge between the Jetson Nano and the probe. Also, it controls the operation of power devices and the system’s power-on and power-off sequences, monitors power requirements, and protects against faults. This module ensures efficient and reliable power management within the system.

Touch display: This is an embedded Graphical User Interface (GUI) module, based on an MCU, which manages the main user interface of the system. This block facilitates a seamless and responsive user experience by connecting the GUI with the underlying application logic. It includes the integration of touch functionality into the overall application, as well as a means to present the information.

### 3.3. System Functionality

**Figure 3:**
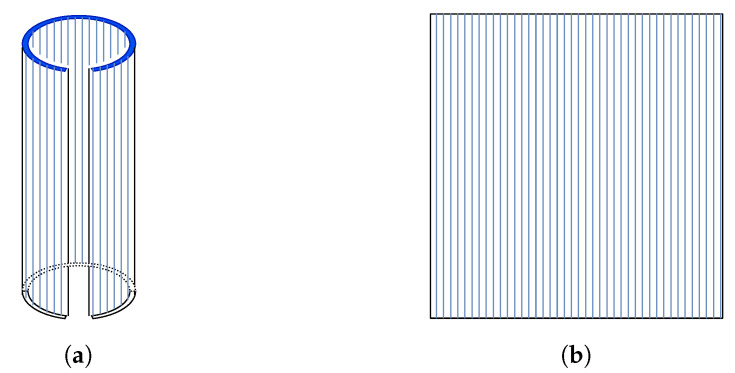
(**a**) Surface sensed during image generation. The arc-shaped path traversed by the transducer is represented by the dark blue arc. (**b**) Acquired image as shown on display. Each scan line formed at each motor step is represented by the light blue vertical lines.

The ultrasound images are generated by the acquisition of consecutive scan lines (A-scan) at discrete step sizes moving along a pre-determined path to build B-scan images. This path is the result of the mechanical motion of the single-element transducer around a fixed rotation axis to prevent the trajectory of an axis being longer than the size of the fontanelle. As shown in probe module in Figure 2, a motor rotates a holder containing the transducer element, placed away from its central axis. This arrangement allows for the transducer to traverse an arc-shaped path (highlighted as a dark blue arc in Figure 3a), describing back and forth displacements in a plane perpendicular to the direction it is pointing to. On each motor step, one scan line is formed (highlighted as light blue vertical lines in Figure 3a) by sending a pulse from the transducer, and then the reflected signals from different depths are recorded. Sequential scan lines are acquired across the body and composed together into a high spatial resolution 2D B-mode image. As the transducer element is moved on the same plane during acquisition, the unrolled image has a rectangular shape, as shown in Figure 3b. This way, the captured ultrasound images are displayed on the monitor.

As shown in the probe module in Figure 2, by means of another motor, the structure that holds the transducer element can be moved along the direction the probe is pointing to. This allows for slightly penetrating (the acquisition window) and, subsequently, deeping the image into the body without increasing the acquisition delay with the consequent depth attenuation. In this way, the area of interest can be aligned with the focal zone for the optimal resolution range of the transducer.

### 3.4. Implementation Details

This heterogeneous computing platform consists of several resources that need to be exploited efficiently to ensure real-time operation and system responsiveness. The main application program (Jetson Nano) is responsible for managing the diverse set of event-driven tasks from the pulser-receiver to the probe, passing through the base, to the touch display. One of the main challenges consists in the global concurrent management of events with minimum latency on the different processors.

Control logic: A multi-threading scheme is implemented in the application software to achieve the required functionality and performance in real time. System execution always begins from the main thread. This thread is effectively used to bootstrap the initialization of three threaded controllers to manage the rest of the modules outside the Jetson Nano (probe through base MCU, pulser-receiver, and touch display). In this way, a specific control structure running on an independent thread can effectively manage a particular instance of the system.

To ensure effective communication between each thread on the application software and the corresponding subsystem, a frame exchange approach is followed. Received frames are processed on a round-robin basis to generate the input conditions to a Finite State Machine (FSM). Specific functionality is provided depending on the current state and transitions to a new state when necessary. State management tracks the current state, allowing the program to respond to user inputs or system events according to the case.

Particularly, the ability to concurrently execute multiple threads on the Jetson Nano requires a complex state machine on the main thread of the application software for managing all the interactions of the data path with the other threads that control the different subsystems. The event-driven nature of embedded systems means that control flow will change often and unpredictably. A successful system should enable transferring control between processes while minimizing the overhead of these transitions. Each threaded controller context does not have direct access to any other, except to the main thread. Data path resources for sharing data with the main thread are provided for a low-latency response to dynamic events. A shared memory model provides a communication channel for passing information between threads.

Data acquisition: The pulser-receiver is configured with one channel, assigning one connector for the emission of the pulse and another connector for the reception of the signal. A preamplifier is used to boost the signal from the transducer on the probe to the pulser-receiver. Communication with the Jetson Nano is established through Ethernet connection. This includes the transference of ultrasound data samples from the pulser-receiver to the Jetson Nano. On the other hand, the Jetson Nano controls the operation of the pulser-receiver and sets its configuration parameters, which ultimately impact the image quality. When the two devices are linked, predefined settings are uploaded to the pulser-receiver during initialization. The configuration parameters are set for the channel used, including the emitter/receiver connectors, receiver gain, acquisition window, pulser parameters, average, designed filters, etc. These parameters can be changed during the operation of the device.

Data acquisition should be triggered at specific intervals aligned with the motor rotation to ensure that data are captured accurately. This is the reason why the pulser-receiver was configured to start acquisitions from an external trigger. The input signal to the driver, which controls when the scan motor takes a step, is passed through the probe cable and connected to the external trigger input on the pulser-receiver. Referred to as *Sync* in Figure 2, it is responsible for triggering the steps of the scan motor at the same time each pulse-echo firing event is started.

**Figure 4:**
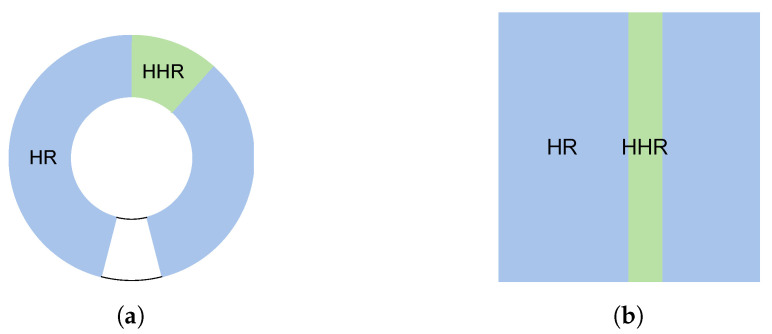
(**a**) HR scan path vs HHR scan path; (**b**) HR img vs HHR img.

Each frame is created from repeated pulse-echos that form scan lines (A-scan) every 1 ms. Ultrasound images are obtained by sets of scan lines. Scan lines are acquired at a 100 MHz sampling frequency. The corresponding sampling period of 10 ns can enable an axial resolution of up to 7.5 μm. The speed of sound in the soft tissue is considered 1500 m/s. An acquisition delay was set to keep the focal zone approximately in the center of the acquisition window. The lateral resolution of the composed image depends on the distance between scan lines. This is controlled by the step size configuration on the scan motor during acquisition. In general, the whole acquisition sequence does not change, including the number of scan lines. First, a wide scan is performed to locate an adequate view of the CSF, hereafter referred to as High Resolution (HR). If an area of interest is found in the CSF, the resolution can be increased by reducing the step size, which will be denoted as Higher Resolution (HHR). This way, the arc path is reduced while maintaining the same number of scan lines, as can be seen in Figure 4. The lateral resolution can range approximately from 15 μm to 2 μm step size.

Signal processing, such as averaging echoes over several cycles and band-pass filtering, was performed. This processing is found useful to enhance the obtained images. The scan lines (A-scan) can be collected as radio-frequency (RF) data acquired by the channel from the pulser-receiver, or as video data, from the envelope obtained after applying a Hilbert transform to this RF signal. When sequential scan lines are acquired across the body and composed together, a high spatial resolution 2D B-mode image can be obtained. Images are displayed on the screen as sequential frames over time. Video image data are often more readily available, particularly in a clinical setting. However, most information is available from the RF ultrasound signal, which typically is not available in commercial machines. Contrast resolution may be enhanced at various stages in the image processing to improve the ability to distinguish between different echo amplitudes of adjacent structures.

Such a data-intensive system allows us to store up to 100 images in a circular buffer, thereby always having the last 100 images captured. At the end of a scan, this image buffer is saved for further analysis. In addition, configuration parameters are recorded for each image in an external file. This process ensures that images are associated with their specific configuration parameters, allowing for detailed tracking and analysis of the conditions under which each image was captured. In general, this is sufficient to allow for an experimental investigation of this novel application.

## 4. Experiments and Results

A definitive demonstration of resolution can be achieved by imaging real features that have already been characterized using a reliable technique, which serves as a ground truth with which to compare. To evaluate the capacity of the proposed solution for imaging microscopic components like WBC, we conducted in vitro experiments that involve imaging particle suspension in ultra pure water (Milli-Q) under different concentrations relevant to meningitis diagnosis (7-, 19-, 69-, and 646-pp/μL). Polystyrene particles with a mean diameter of 7 μm were used to mimic approximately the dimensions of white blood cells [45,46].

**Figure 5:**
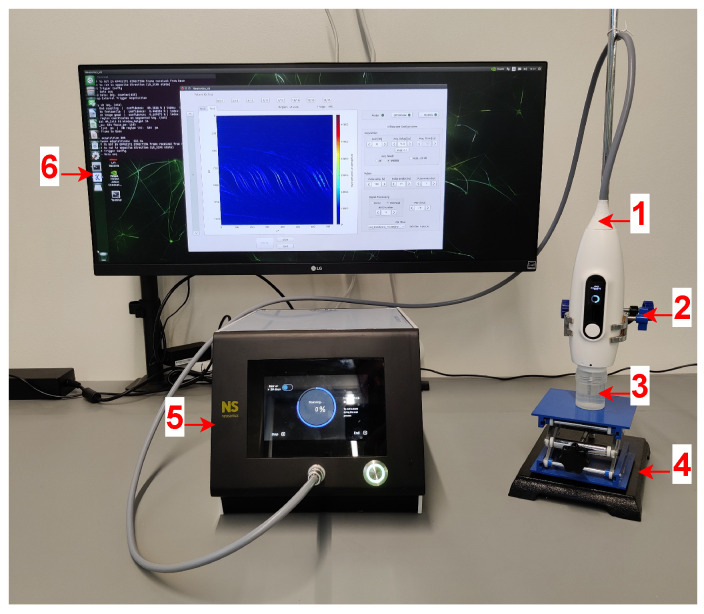
Experimental setup composed of (1) ultrasound probe, (2) bracket, (3) sample container, (4) elevating platform, (5) base module, and (6) external monitor.

Two specific setups were mounted to conduct the experimental tests. The basic idea is to go from a simpler setup based on a container with the sample to an anatomy-based phantom of a neonatal head (also embodied in a container). As shown in Figure 5, the probe is held with a bracket with 2D versatile movement that allows for the transducer to be oriented vertically downwards against the sample container. The sample container is placed on an elevating platform that allows it to be brought close to the transducer. At a high resolution, any significant human movement compromises the quality of the acquired image. Once the sample container is fixed at a contact distance to the transducer, depth adjustment is mechanically controlled in the probe by the motors.

**Figure 6:**
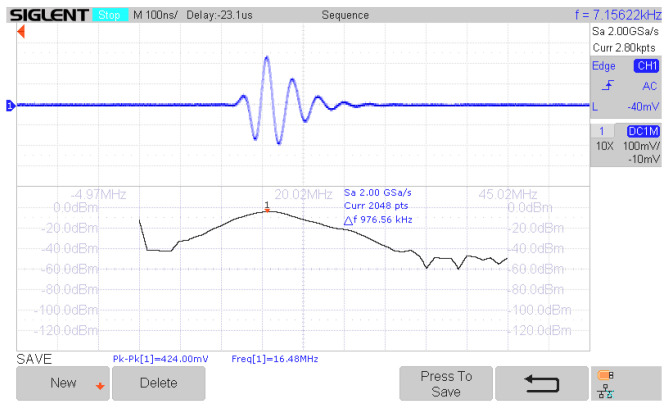
Pulse-echo impulse response and spectrum of the 20 MHz transducer measured with a SIGLENT oscilloscope.

Before the experiments, the pulse-echo ultrasound configuration was characterized using a flat brass reflector placed in front of the transducer, being both immersed in a water bath. The distance between the transducer and the reflector is adjusted around the nominal focal distance to obtain the maximum intensity. Figure 6 shows the pulse-echo response of the 20 MHz transducer measured with an oscilloscope from SIGLENT Technologies (Shenzhen, China).

For both experimental setups, batches of 100 images were acquired for the different WBC concentrations. These samples were characterized at the analysis laboratory in Barcelona Scientific Park (Barcelona, Spain) using the Fuchs Rosenthal method for concentrations lower than 200 pp/μL and the Neubauer method for concentrations equal or higher than this value. The experiments were performed just after sample preparation in order to prevent the particles from settling on the bottom or walls of the container.

### 4.1. Setup 1

For the first setup, the probe was dipped into an ultra pure water suspension with 7 μm diameter particles with the concentrations mentioned above. The probe was introduced in the container at a minimum depth such that the transducer emitting face was in direct contact with the water. Mechanical alignment was performed via the elevating platform while receiving real-time image feedback from the application software, ensuring that the focal region is inside the area of interest in the liquid.

**Figure 7:**
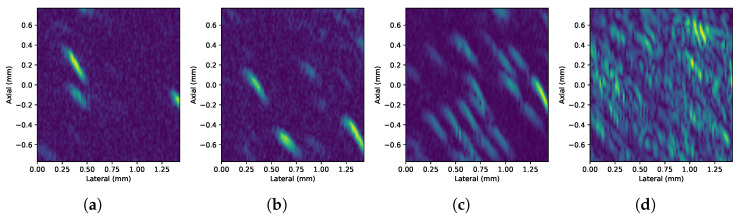
Detail of the HR B-mode images (video) obtained with setup 1 on 7 μm particles’ suspension under (**a**) 7-, (**b)** 19-, (**c**) 69-, and (**d**) 646-pp/μL concentrations, respectively, from left to right.

**Figure 8:**
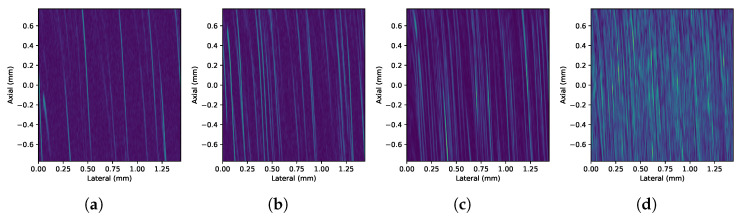
Detail of the HHR B-mode images (video) obtained with setup 1 on 7 μm particles’ suspension under (**a**) 7-, (**b**) 19-, (**c**) 69-, and (**d**) 646-pp/μL concentrations, respectively, from left to right.

Figure 7 shows representative frames of experimental HR B-mode images (video) obtained from setup 1 for every concentration. Similarly, Figure 8 shows the images corresponding to the HHR-mode acquisitions.

### 4.2. Setup 2

The second setup is a phantom based on the anatomy of a neonatal head, embodied in a container as in the previous case. This emulates a tube section from the fontanelle cap to the cortex of the brain. The fontanelle was modeled using a 4 mm thick tissue-mimicking layer made of polyvinyl alcohol (PVA)-based hydrogel [47]. The pulse-echo total attenuation of this layer at 20 MHz is 6.3 dB, similar to the real fontanelle [14]. The zone between the fontanelle cap and the cortex corresponds to the CSF simulated with the sample solution. A coupling gel is applied on the fontanelle cap to facilitate energy transfer. The probe is then lowered into the coupling gel, simulating the proposed mode of operation. Due to the physical fragility of the phantom, the transducer cannot contact the fontanelle during scanning but scans through the thin gel layer between the transducer head and the fontanelle cap.

It is important to locate the CSF zone inside this model, where the particles are located. To this end, the transducer is moved mechanically using the depth motor in the probe until aligning the area of interest with the focal zone in the center of the image.

**Figure 9:**
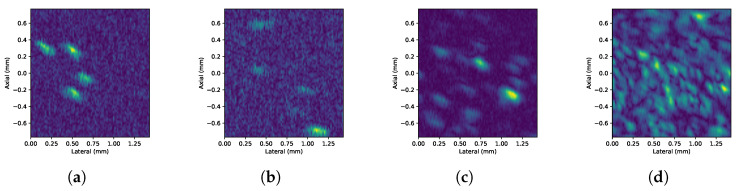
Detail of the HR B-mode images (video) obtained with setup 2 on 7 μm particles’ suspension under (**a**) 7-, (**b**) 19-, (**c**) 69-, and (**d**) 646-pp/μL concentrations, respectively, from left to right.

**Figure 10:**
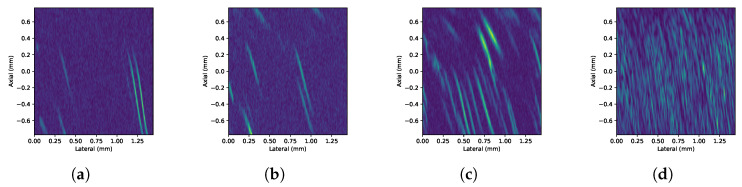
Detail of the HHR B-mode images (video) obtained with setup 2 on 7 μm particles’ suspension under (**a**) 7-, (**b**) 19-, (**c**) 69-, and (**d**) 646-pp/μL concentrations, respectively, from left to right.

Figure 9 shows representative frames of experimental HR B-mode images (video) obtained with setup 2 for 7-, 19-, 69-, and 646-pp/μL concentrations, respectively. Similarly, Figure 10 shows the images corresponding to HHR-mode acquisitions.

## 5. Discussion

The ultimate measurement of resolution in this application is to distinguish particles from liquid. The experimental results show that the implementation proposed can attain 7 μm particles. In the horizontal band running through the center of the image, where the focal zone is located, the bright traces of the particles can be clearly distinguished from the background. This focusing effect becomes more evident as the concentration increases. As the concentration increases, it can also be observed that traces of nearby particles can overlap and appear as single particles or clusters in the image. The superposition of echoes is caused by the proximity between particles beyond the spatial resolution. This effect is due to the fact that the echoes of the particles are larger than the particles themselves. This is a consequence of the wavelength being greater than the size of the particles [14].

**Figure 11:**
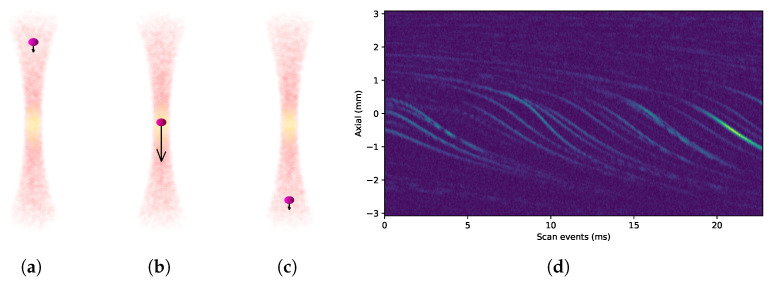
Difference in the pressure effect of the ultrasound beam on the speed of a particles depending on its location: (**a**) above the focal area, (**b**) in the focal area, and (**c**) below the focal area. (**d**) Since ultrasound images are generated using consecutive scan lines, the motion of particles is translated into the image as the characteristic sigmoid-shaped traces. This location-dependent pressure effect is maximized when multiple scans are consecutively shot without moving the scanner head (M-mode, extreme case).

It is important to notice that the pulse firings affects the path of the particles as shown in their flow patterns on Figure 8a–d and Figure 10a–d for HHR scans on both experiments. Figure 11a–c depicts how particles are actually pushed down by each repeated pulse-echo used to form a scan line. Since ultrasound images are generated using consecutive scan lines, the motion of particles is translated into the image as the characteristic sigmoid-shaped traces observed in Figure 11d and others above.

This movement is due to the ultrasound pressure field produced by the focused transducer that is stronger in the focal zone and may end up moving a particle out of the focal zone after several acquisitions. On the other hand, these movements push new particles to the focal zone. The sigmoid effect at the ends of the traces is due to the fact that, at the ends of the focal zone, the particle receives less pressure, so the displacement effect is less pronounced when composing the image. The acoustic origin of this force is observed when the pulse energy and the pulse repetition frequency (PRF) are varied in the vicinity of the particle. This effect is evident in HHR scanning, where the scan lines are acquired closer together to increase the lateral resolution. When several acquisitions are obtained and averaged to get every single A-scan at any given position, this effect is even more noticeable when the PRF is increased. The extreme case is achieved by obtaining acquisitions in the same position without moving the scan motor (M-mode), as shown in Figure 11d.

As mentioned in Section 3.3, this imaging method acquires scan lines to create the image by sending multiple pulses sequentially. Particles move during the data acquisition process. However, the temporal resolution is sufficient to track the resulting particle pattern in the images with lateral coherence.

## 6. Conclusions

Direct detection of 7 μm particles in water suspensions was achieved using a novel high-frequency ultrasound platform at 20 MHz. Experimental measurements were conducted to characterize particles in different setups. This experiments provided valuable information on the concentration, size, and unique flow pattern of such particles that can be directly aligned with those present in white blood cells inside the CSF.

The developed system allows for acquiring images with high-resolution, providing excellent clarity and detail. The high temporal resolution provided by this imaging method has enabled a smooth visualization of particle flow inside the experimental setup, which probes the temporal resolving power of this imaging paradigm in depicting flow motion. Its advanced hardware components and optimized software implementation ensure efficient and reliable image acquisition.

This represents a promising approach to the detection of infant meningitis, offering advantages in terms of accessibility, safety, and ease of use. Further research and development efforts are warranted to refine the technique, validate its clinical utility, and ultimately improve outcomes for neonates at risk of meningitis. The capability of the system to acquire high-resolution images paves the way for enhanced analysis and a deeper understanding of ultrasound data.

## 7. Patents

Segura, L. E., Graullera, O. M., SHUKLA, S. K., Montero, F., Jimenez, J., Butterworth, I. R., ⋯ and Gonzalez, C. C. (2020). U.S. Patent No. 10,639,012. Washington, DC: U.S. Patent and Trademark Office [48].

## Data Availability

The data that support the findings of this study are available from the corresponding author, M.N., upon reasonable request.

## References

[1] Koelman D.L., Van Kassel M.N., Bijlsma M.W., Brouwer M.C., Van De Beek D., Van Der Ende A. (2021). Changing epidemiology of bacterial meningitis since introduction of conjugate vaccines: 3 decades of national meningitis surveillance in The Netherlands. Clin. Infect. Dis..

[2] Zunt J.R., Kassebaum N.J., Blake N., Glennie L., Wright C., Nichols E., Abd-Allah F., Abdela J., Abdelalim A., Adamu A.A. (2018). Global, regional, and national burden of meningitis, 1990–2016: A systematic analysis for the Global Burden of Disease Study 2016. Lancet Neurol..

[3] Hersi K., Gonzalez F.J., Kondamudi N.P. (2023). Meningitis. StatPearls.

[4] Qu C., Wang Y., Wang X., He R., Cao H., Liu B., Zhang H., Zhang N., Lai Z., Dai Z. (2023). Global burden and its association with socioeconomic development status of meningitis caused by specific pathogens over the past 30 years: A population-based study. Neuroepidemiology.

[5] Jane L.A., Wray A.A. (2023). Lumbar puncture. StatPearls.

[6] Tunkel A.R., van de Beek D., Scheld W.M. (2014). Acute meningitis. Principles and Practice of Infectious Diseases.

[7] Mount H.R., Boyle S.D. (2017). Aseptic and bacterial meningitis: Evaluation, treatment, and prevention. Am. Fam. Physician.

[8] Glatstein M.M., Zucker-Toledano M., Arik A., Scolnik D., Oren A., Reif S. (2011). Incidence of traumatic lumbar puncture: Experience of a large, tertiary care pediatric hospital. Clin. Pediatr..

[9] Zainel A., Mitchell H., Sadarangani M. (2021). Bacterial meningitis in children: Neurological complications, associated risk factors, and prevention. Microorganisms.

[10] van der Flier M. (2021). Neonatal meningitis: Small babies, big problem. Lancet Child Adolesc. Health.

[11] Khalessi N., Afsharkhas L. (2014). Neonatal meningitis: Risk factors, causes, and neurologic complications. Iran. J. Child Neurol..

[12] Elvira L., Vera P., Cañadas F.J., Shukla S.K., Montero F. (2016). Concentration measurement of yeast suspensions using high frequency ultrasound backscattering. Ultrasonics.

[13] Jimenez X., Shukla S.K., Ortega I., Illana F.J., Castro-González C., Marti-Fuster B., Butterworth I., Arroyo M., Anthony B., Elvira L. (2016). Quantification of very low concentrations of leukocyte suspensions in vitro by high-frequency ultrasound. Ultrasound Med. Biol..

[14] Elvira L., Rodríguez A.I., Fernández A., Durán C., Romero M.P., Pose-Díez-de-la Lastra A., Bassat Q., Jiménez J. (2020). A New Methodology for the Assessment of Very Low Concentrations of Cells in Serous Body Fluids Based on the Count of Ultrasound Echoes Backscattered From Cells. IEEE Trans. Ultrason. Ferroelectr. Freq. Control.

[15] Elvira L., Fernández A., León L., Ibáñez A., Parrilla M., Martínez Ó., Jiménez J. (2023). Evaluation of the Cell Concentration in Suspensions of Human Leukocytes by Ultrasound Imaging: The Influence of Size Dispersion and Cell Type. Sensors.

[16] Dudink J., Jeanne Steggerda S., Horsch S. (2020). State-of-the-art neonatal cerebral ultrasound: Technique and reporting. Pediatr. Res..

[17] Ecury-Goossen G.M., Camfferman F.A., Leijser L.M., Govaert P., Dudink J. (2015). State of the art cranial ultrasound imaging in neonates. JoVE J. Vis. Exp..

[18] Pichardo S., Sin V.W., Hynynen K. (2010). Multi-frequency characterization of the speed of sound and attenuation coefficient for longitudinal transmission of freshly excised human skulls. Phys. Med. Biol..

[19] Grogan S.P., Mount C.A. (2021). Ultrasound Physics and Instrumentation. StatPearls.

[20] Couture O., Hingot V., Heiles B., Muleki-Seya P., Tanter M. (2018). Ultrasound localization microscopy and super-resolution: A state of the art. IEEE Trans. Ultrason. Ferroelectr. Freq. Control.

[21] Christensen-Jeffries K., Couture O., Dayton P.A., Eldar Y.C., Hynynen K., Kiessling F., O’Reilly M., Pinton G.F., Schmitz G., Tang M.X. (2020). Super-resolution ultrasound imaging. Ultrasound Med. Biol..

[22] Couture O., Besson B., Montaldo G., Fink M., Tanter M. (2011). Microbubble ultrasound super-localization imaging (MUSLI). Proceedings of the 2011 IEEE International Ultrasonics Symposium.

[23] Deffieux T., Demené C., Tanter M. (2021). Functional ultrasound imaging: A new imaging modality for neuroscience. Neuroscience.

[24] Bar-Zion A., Tremblay-Darveau C., Solomon O., Adam D., Eldar Y.C. (2016). Fast vascular ultrasound imaging with enhanced spatial resolution and background rejection. IEEE Trans. Med. Imaging.

[25] Pang B., Ta D., Liu X. (2023). A super-resolution ultrasound imaging method based on active-modulated super-resolution optical fluctuation imaging. Proceedings of the 2023 45th Annual International Conference of the IEEE Engineering in Medicine & Biology Society (EMBC).

[26] Ilovitsh T., Ilovitsh A., Foiret J., Fite B.Z., Ferrara K.W. (2018). Acoustical structured illumination for super-resolution ultrasound imaging. Commun. Biol..

[27] Talapin D.V., Murray C.B. (2005). PbSe nanocrystal solids for n-and p-channel thin film field-effect transistors. Science.

[28] Lanoy M., Pierrat R., Lemoult F., Fink M., Leroy V., Tourin A. (2015). Time reversal sub-wavelength focusing in bubbly media. arXiv.

[29] Fink M., Tanter M. (2010). Multiwave imaging and super resolution. Phys. Today.

[30] Strohm E.M., Moore M.J., Kolios M.C. (2016). High resolution ultrasound and photoacoustic imaging of single cells. Photoacoustics.

[31] Kolios M.C., Taggart L., Baddour R., Foster F., Hunt J., Czarnota G., Sherar M. (2003). An investigation of backscatter power spectra from cells, cell pellets and microspheres. Proceedings of the IEEE Symposium on Ultrasonics.

[32] Baddour R.E., Sherar M., Hunt J., Czarnota G., Kolios M.C. (2005). High-frequency ultrasound scattering from microspheres and single cells. J. Acoust. Soc. Am..

[33] Baddour R.E., Kolios M.C. (2007). The fluid and elastic nature of nucleated cells: Implications from the cellular backscatter response. J. Acoust. Soc. Am..

[34] Han A., Abuhabsah R., Blue J.P., Sarwate S., O’Brien W.D. (2011). Ultrasonic backscatter coefficient quantitative estimates from high-concentration Chinese hamster ovary cell pellet biophantoms. J. Acoust. Soc. Am..

[35] Chen S.H., Lin Y.H., Li W.T., Wang S.H., Huang C.C. (2012). Estimation of cell concentration using high-frequency ultrasonic backscattering. J. Med. Biol. Eng..

[36] Tigner A., Ibrahim S.A., Murray I.V. (2023). Histology, White Blood Cell. StatPearls.

[37] Mattoon J.S., Nyland T., Mattoon J.S., Nyland T.G. (2015). Fundamentals of diagnostic ultrasound. Small Anim Diagnostic Ultrasound Mattoon.

[38] Aldrich J.E. (2007). Basic physics of ultrasound imaging. Crit. Care Med..

[39] Shastri S.K., Rudresh S., Anand R., Nagesh S., Seelamantula C.S., Thittai A.K. (2020). Axial super-resolution in ultrasound imaging with application to non-destructive evaluation. Ultrasonics.

[40] Ng A., Swanevelder J. (2011). Resolution in ultrasound imaging. Contin. Educ. Anaesthesia Crit. Care Pain.

[41] Cootney R.W. (2001). Ultrasound imaging: Principles and applications in rodent research. ILAR J..

[42] Rupitsch S.J., Rupitsch S.J. (2019). Piezoelectric Ultrasonic Transducers. Piezoelectric Sensors and Actuators: Fundamentals and Applications.

[43] Moran C.M., Thomson A.J. (2020). Preclinical ultrasound imaging—a review of techniques and imaging applications. Front. Phys..

[44] Oumer M., Tazebew A., Alemayehu M. (2021). Anterior fontanel size among term newborns: A systematic review and meta-analysis. Public Health Rev..

[45] Lee J.H., Boning D.S., Anthony B.W. (2018). Measuring the absolute concentration of microparticles in suspension using high-frequency B-mode ultrasound imaging. Ultrasound Med. Biol..

[46] Strohm E.M., Gnyawali V., Sebastian J.A., Ngunjiri R., Moore M.J., Tsai S.S., Kolios M.C. (2019). Sizing biological cells using a microfluidic acoustic flow cytometer. Sci. Rep..

[47] Elvira L., Durán C., Higuti R.T., Tiago M.M., Ibáñez A., Parrilla M., Valverde E., Jiménez J., Bassat Q. (2019). Development and characterization of medical phantoms for ultrasound imaging based on customizable and mouldable polyvinyl alcohol Cryogel–Based materials and 3-D printing: Application to high-frequency cranial ultrasonography in infants. Ultrasound Med. Biol..

[48] Segura L.E., Graullera O.M., SHUKLA S.K., Montero F., Jimenez J., Butterworth I.R., Anthony B., Haeseon J.L., Gonzalez C.C. (2020). Method for Detecting Circulating Cells in Superficial Body Fluids. US Patent.

